# A Novel Saliva-Based miRNA Signature for Colorectal Cancer Diagnosis

**DOI:** 10.3390/jcm8122029

**Published:** 2019-11-20

**Authors:** Óscar Rapado-González, Blanca Majem, Ana Álvarez-Castro, Roberto Díaz-Peña, Alicia Abalo, Leticia Suárez-Cabrera, Antonio Gil-Moreno, Anna Santamaría, Rafael López-López, Laura Muinelo-Romay, María Mercedes Suarez-Cunqueiro

**Affiliations:** 1 Department of Surgery and Medical-Surgical Specialties, Medicine and Dentistry School, Universidade de Santiago de Compostela (USC), 15782 Santiago de Compostela, Spain; oscar.rapado@rai.usc.es; 2 Liquid Biopsy Analysis Unit, Translational Medical Oncology (Oncomet), Health Research Institute of Santiago (IDIS), 15706 Santiago de Compostela, Spain; roberdp78@gmail.com (R.D.-P.); Alicia.Abalo.Pineiro@sergas.es (A.A.); 3 Centro de Investigación Biomédica en Red de Cáncer (CIBERONC), Instituto de Salud Carlos III, 28029 Madrid, Spain; antonioimma@yahoo.es (A.G.-M.); rafa.lopez.lopez@gmail.com (R.L.-L.); 4 Cell Cycle and Cancer Laboratory, Group of Biomedical Research in Urology, Vall Hebron Research Institute (VHIR), Universitat Autònoma de Barcelona (UAB), 08035 Barcelona, Spain; b.majem@gmail.com (B.M.); leticia.suarez@vhir.org (L.S.-C.); anna.santamaria@vhir.org (A.S.); 5 Cell Death Regulation Group, Oncobell Program, Bellvitge Biomedical Research Institute (IDIBELL), L’Hospitalet de Llobregat, 08908 Barcelona, Spain; 6 Medical Digestive Service, Complexo Hospitalario Universitario de Santiago de Compostela (SERGAS), 15706 Santiago de Compostela, Spain; anacastro189@gmail.com; 7 Faculty of Health Sciences, Universidad Autónoma de Chile, Talca 3460000, Chile; 8 Unit of Gynecologic Oncology, Department of Obstetrics and Gynecology, Hospital Universitario Vall d’Hebron, 08035 Barcelona, Spain; 9 Translational Medical Oncology (Oncomet), Health Research Institute of Santiago (IDIS), Complexo Hospitalario Universitario de Santiago de Compostela (SERGAS), 15706 Santiago de Compostela, Spain; 10 Oral Sciences, Health Research Institute of Santiago de Compostela (IDIS), 15706 Santiago de Compostela, Spain

**Keywords:** liquid biopsy, saliva, colorectal cancer, microRNA, circulating biomarkers, epigenomics

## Abstract

Salivary microRNAs (miRNAs) are of high interest as diagnostic biomarkers for non-oral cancer. However, little is known about their value for colorectal cancer (CRC) detection. Our study aims to characterize salivary miRNAs in order to identify non-invasive markers for CRC diagnosis. The screening of 754 miRNAs was performed in saliva samples from 14 CRC and 10 healthy controls. The differential expressed miRNAs were validated by RT-qPCR in 51 CRC, 19 adenomas and 37 healthy controls. Receiver operating characteristic (ROC) curves and logistic regression models were performed to analyze the clinical value of these miRNAs. Twenty-two salivary miRNAs were significantly deregulated in CRC patients vs. healthy individuals (*p* < 0.05) in the discovery phase. From those, five upregulated miRNAs (miR-186-5p, miR-29a-3p, miR-29c-3p, miR-766-3p, and miR-491-5p) were confirmed to be significantly higher in the CRC vs. healthy group (*p* < 0.05). This five-miRNA signature showed diagnostic value (72% sensitivity, 66.67% specificity, AUC = 0.754) to detect CRC, which was even higher in combination with carcinoembryonic antigen (CEA) levels. Overall, after the first global characterization of salivary miRNAs in CRC, a five-miRNA panel was identified as a promising tool to diagnose this malignancy, representing a novel approach to detect cancer-associated epigenetic alterations using a non-invasive strategy.

## 1. Introduction

Colorectal cancer (CRC) is the third most commonly diagnosed cancer in men and the second in women worldwide. Regarding mortality data, it represents the fourth and the third cause of cancer death in men and women, respectively [1]. In 2030, the global burden CRC is expected to increase by 60% to over 2.2 million new cases and 1.1 million deaths [2]. Approximately 50% of CRC patients present metastasis at diagnosis or develop it during the course of the disease [3]. Currently, CRC diagnostic procedures are invasive or show low sensitivity and specificity, and thus research efforts are directed towards the search for less invasive biomarkers with higher accuracy to detect patients.

Recently, a growing number of researchers have reported the potential of microRNAs (miRNAs) as diagnostic biomarkers in CRC [4,5]. MiRNAs are small, noncoding RNA molecules of 18–22 nucleotides that act as regulators of gene expression, interacting mainly with messenger RNA. MiRNAs expression can be deregulated in the pathogenesis and development of human cancer, and they can act as oncogenes or tumor suppressors [6]. Several miRNAs are released by cells [7] in physiological and pathological conditions into the extracellular space and can then be incorporated, free or encapsulated, into the bloodstream and other body fluids, such as saliva, urine, and cerebrospinal fluid [8], as “circulating miRNAs”—they have high stability due to their small size and their association with extracellular vesicles and RNA-binding proteins, such as argonaute-2 or high-density lipoproteins [9]. Due to their unique characteristics, miRNAs detected in liquid biopsies are promising biomarkers for cancer diagnosis, with a clear potential application for patients management in daily clinical practice. To date, several authors have described circulating miRNAs in blood with high diagnostic value and prognostic impact in CRC [10]. 

Ongoing scientific evidence in the salivary research field reinforces the interest of this biofluid as a source of potential biomarkers, identified in different salivary ‘-omics’ including the genome, the microbiome, the epigenome, the transcriptome, the proteome and the metabolome [11]. In this sense, saliva presents many advantages as a diagnostic tool over blood or tissue. The sample collection is even less invasive than blood and easy to store and transport [12]. In addition, saliva can be collected in shorter intervals than other diagnostic samples in order to provide updated tracking and patient follow-up without involving any discomfort for the patient. Aberrant expression profiles of salivary miRNAs have been detected in different types of cancer, showing their power as a discriminatory clinical method [13]. Unlike other tumors, only salivary miR-21 was analyzed in CRC by Sazanov et al. [14], finding miR-21 expression significantly higher in the saliva of CRC patients compared to healthy controls. 

Based on these findings, we have analyzed miRNAs profiles in a large cohort of saliva samples from CRC patients, adenomas and healthy individuals. With this approach, we were able to identify and validate salivary miRNAs with discriminatory value in detecting CRC.

## 2. Experimental Section

### 2.1. Study Subjects and Sample Collection

This study was approved by the Galician Ethics Committee of Clinical Research (Ref. No. 2013/371) and written informed consent was obtained from all participants involved in this study at the time of recruitment. The methods were performed in accordance with relevant guidelines and regulations, following the recommendations of the PRoBE design [15]. This study complied the principles outlined in the Declaration of Helsinki [16]. 

One hundred and seven saliva samples from 51 CRC patients, 19 adenomas and 37 healthy individuals were collected from 2014 to 2018 at Oncology and Digestive Services from Complexo Hospitalario Universitario of Santiago de Compostela (SERGAS) in Galicia, Spain. All CRC and adenoma cases were confirmed by histopathological analysis of the corresponding tissue biopsy. Tumor stage in CRC patients was determined in accordance with the Tumor-Node-Metastasis (TNM)-based staging system promulgated by the American Joint Commission on Cancer (AJCC). None of the patients under study received chemotherapy or radiotherapy before sample collection. Eight patients underwent resective surgery before sample collection. Patients with a history of familial adenomatous polyposis, hereditary non-polyposis CRC, inflammatory bowel disease or diagnosis of other cancer in the last 5 years were excluded from this study. This study was designed as two phases: a discovery phase including 24 saliva samples (10 healthy controls and 14 CRC, including 7 non-metastatic and 7 metastatic patients) and a validation phase comprising 107 saliva samples (37 healthy controls, 19 adenomas and 51 CRC), including those used for the discovery phase (Figure S1). Clinicopathologic characteristics and lifestyle habits are summarized in Table S1. The detailed saliva collection and processing can be found in Supplementary Methods.

### 2.2. Saliva RNA Extraction 

Total RNA containing miRNAs was extracted from 500 μL of saliva using miRNeasy extraction Micro Kit (Qiagen, Hilden, Germany) according to the manufacturer’s protocol with some modifications (Supplementary Methods). The concentration and quality of total RNA were analyzed using the Agilent 2100 Bioanalyzer and Quantus™ Fluorometer (Promega).

### 2.3. Salivary MiRNA Discovery Profiling with TaqMan Low-Density Arrays

MiRNA expression profiling was performed using TaqMan^®^ Low-Density Human MicroRNA Arrays (TLDA, Applied Biosystems, Foster City, California, USA). Each TLDA card detects 384 probes including 377 human miRNAs, 3 endogenous control assays and 1 negative control assay. Together, the 2 array cards (A and B Cards v3.0) can detect 754 mature miRNAs present in miRBase v14. This miRNA microarray assay was performed using 3 µL (1–350 ng) of total RNA from each sample, according to the manufacture’s protocol (Supplementary Methods). 

### 2.4. MiRNA Expression Analysis Using RT-qPCR 

Expression levels of candidate miRNAs were validated by miRNA TaqMan RT-qPCR assays (Applied Biosystems) according to the manufacturer´s protocol (Supplementary Methods). Relative gene expression was determined by the 2^-∆∆Ct^ method [17] and normalized with miR-193b-3p, which showed high stability among our samples. The selection of this internal control for quantification of salivary miRNA was described in Supplementary Methods (Table S2 and Figure S2).

### 2.5. Functional Enrichment Analysis

Pathway enrichment analysis was performed using the DNA Intelligent Analysis (DIANA)-miRPath v3.0 software [18] to determine the miRNA regulatory roles and to identify the significant Kyoto Encyclopedia of Genes and Genomes (KEGG) molecular pathways. The algorithm miRTarBase v7.0 was used to identify the validated targets miR-186-5p, miR-29a-3p, miR-29c-3p, miR-766-3p and miR-491-5p. We selected the “pathways union” option of the miRPath software. The threshold (*p* < 0.05) and the false discovery rate (FDR) (*p* < 0.05) were calculated by Fisher´s exact test.

### 2.6. Statistical Analysis

All statistical analyses with IBM SPSS Statistics 20 and graphs were generated using GraphPad Prism 5.0 (GraphPad Software, Inc., San Diego, California, USA). Hierarchical clustering was performed using the R environment (http://www.r-project.org/) and a heatmap was generated using a function of heatmap.2 in gplots package. Two-tailed Mann–Whitney U test or the Kruskal–Wallis test was used to evaluate the differential expression of salivary miRNAs. Multivariate logistic regression analyses were performed to establish the best miRNA panel to discriminate CRC from healthy individuals and those stage IV CRC with bad evolution of the disease (defined as progression or death within the 10 months after sample collection). The following formulas were generated after the logistic regression to provide a score for classifying patients vs. healthy controls and good/bad prognosis stage IV CRC patients: score for diagnosis = −9 − (1.4 * expression_miR-29a-3p_) + (0.6 * expression_miR1-86-5p_) + (2.1 * expression_miR-29c-3p_) − (0.38 * expression_miR-491-5p_) + (0.01 * expression_miR-766-3p_); risk-score for prognosis = 1.09 + (5.4 * expression_miR-29a-3p_) − (1.4 * expression_miR-186-5p_) − (5.7 * expression_miR-29c-3p_) + (1.03 * expression_miR-491-5p_) + (0.39 * expression_miR-766-3p_). Receiver operating characteristic (ROC) curves were constructed, and area under the ROC curve (AUC) with 95% of confidence intervals (CIs) was obtained to evaluate the diagnostic accuracy of individual salivary miRNAs and the miRNA panel. Internal validation with the bootstrap method was used to adjust the overfitting. A total of 2000 random samples with replacement were generated. The AUC and the 95% CIs for the sensitivity and specificity were estimated using the pROC package in R software. Cut-off selection was made based on the value that provided the highest sensitivity and specificity to discriminate healthy controls vs. CRC patients and CRC patients with good vs. bad outcomes. For survival analyses Univariate/Multivariate Cox Regression was applied together with the Kaplan–Meier curves and the Log-Rank test. Spearman correlations were performed to determine the relationship among the different miRNAs and between each miRNA and the carcinoembryonic antigen (CEA) blood levels. *p*-value < 0.05 was set as the level of statistical significance. 

## 3. Results

### 3.1. Salivary miRNA Expression Profiling Identified a Specific Expression Pattern in CRC

During the discovery phase, 754 miRNAs were profiled in saliva samples from 14 CRC patients and 10 healthy controls. From all miRNAs included in the panel, 31 miRNAs (4.11%) were detected in all samples, 194 miRNAs (25.73%) were detected in >50% and 294 miRNAs (38.99%) were not present in any salivary sample. To detect those miRNAs characterizing CRC patients, we selected miRNAs present in at least 70% of patients, being significantly differentially expressed in patients (metastatic and/or non-metastatic) vs. healthy controls with a minimum fold change of 1.5 (up or down). Following these criteria, 22 miRNAs were found significantly deregulated in the saliva of patients compared to healthy controls. Expression levels of 20 miRNAs were found upregulated while only two were downregulated. The Venn diagram (Figure S3) shows differentially expressed miRNAs in cancer and controls taking into account the tumor stage. Thus, 18 miRNAs were found differentially expressed in non-metastatic patients, two in metastatic patients and six in both non-metastatic and metastatic patients.

### 3.2. A Panel of Salivary miRNAs Was Validated for CRC 

**Figure 1:**
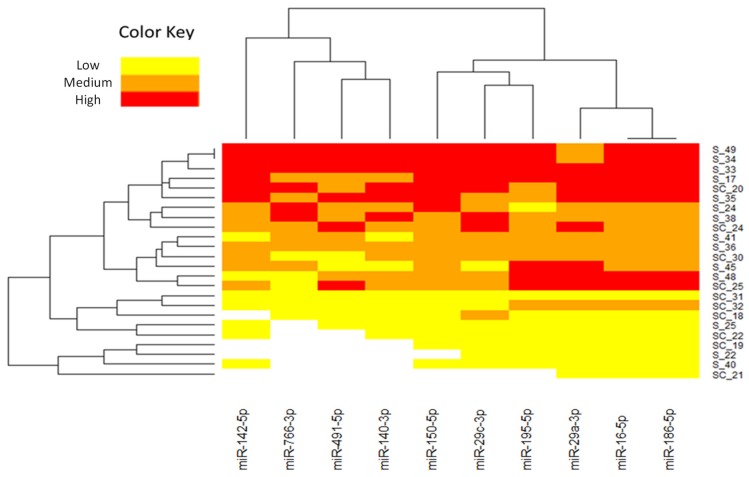
Hierarchical clustering heatmap of 10 miRNAs selected for the validation phase with a > 1.5-fold change difference (*p* < 0.05) between CRC patients and healthy controls. Each row represents one sample and each column represents the expression profile of a single miRNA. The relative miRNA expression changes are expressed by three colors from red to yellow, as shown at the top, where red represents the high expression, orange medium expression, and yellow low expression. The miRNA clustering tree is shown on the top and the sample clustering dendrogram is presented on the left. S1–S12 correspond to salivary samples of CRC patients and SC1–SC10 correspond to salivary samples of healthy controls. Note: miRNAs, microRNAs; CRC, colorectal cancer.

**Figure 2:**
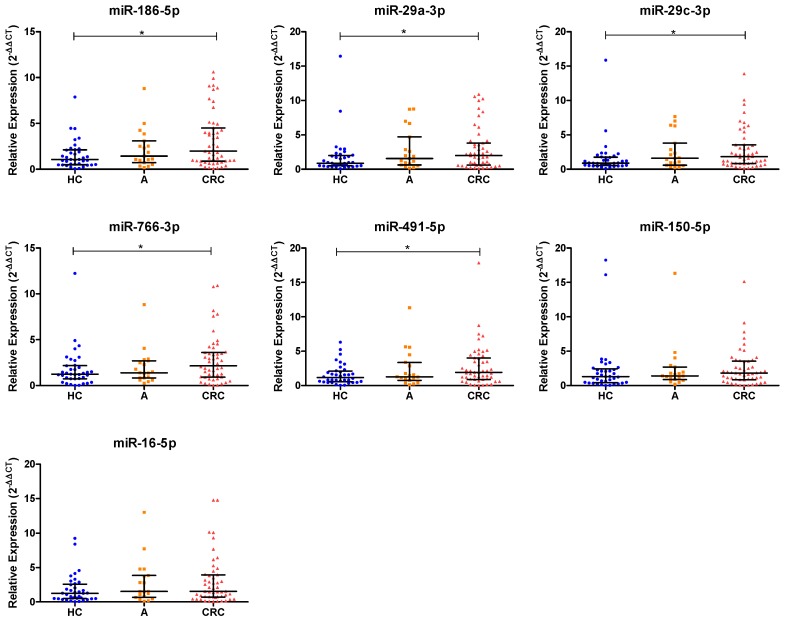
Salivary miRNA expression levels in healthy controls, adenomas and CRC patients in the validation phase of the study. The relative expression levels of selected salivary miRNAs were normalized to endogenous control (miR-193b-3p). The two-tailed Mann–Whitney U test was performed to examine the difference between groups of seven miRNAs (* *p* < 0.05). Note: HC, healthy controls; A, adenomas; CRC, colorectal cancer; miRNAs, microRNAs.

Ten miRNAs (miR-186-5p, miR-29a-3p, miR-766-3p, miR-16-5p, miR-195-5p, miR-140-3p, miR-142-5p, miR-29c-3p, miR-150-5p and miR-491-5p) were chosen from a total of the 22 differential miRNAs based on their fold change and their previous description as miRNAs expressed in CRC tumors (Figure 1). To verify whether the selected miRNAs were upregulated in saliva, we first examined their levels in a set of 30 CRC and 15 healthy individuals. Significant differences were found in seven miRNAs (miR-186-5p, miR-29a-3p, miR-766-3p, miR-16-5p, miR-29c-3p, miR-150-5p and miR-491-5p). These miRNAs were further validated in the full large-scale set of saliva samples from a total of 107 CRC, 19 adenomas and 37 healthy individuals. As shown in Figure 2, after the complete analysis, we found that salivary levels of miR-186-5p (*p* = 0.0136), miR-29a-3p (*p* = 0.0376), miR-29c-3p (*p* = 0.0112), miR-766-5p (*p* = 0.0381) and miR-491-5p (*p* = 0.0366) were statistically significantly different between the CRC and healthy groups. Salivary expression levels of miR-16-5p and miR-150-5p were higher in CRC than in healthy individuals, but no significant differences were obtained. In addition, the expression levels of validated miRNAs were also increased in patients with adenomas compared to healthy controls, however no significant differences were found. 

**Figure 3:**
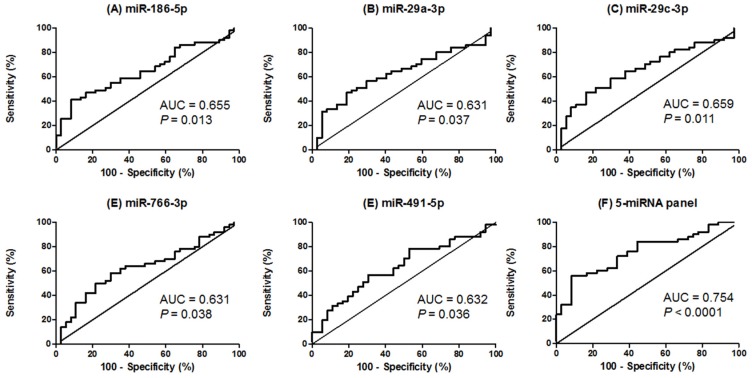
ROC curve analysis using salivary miRNA levels to discriminate CRC patients from healthy individuals. (**A**) Salivary miR-186-5p levels yielded an AUC value of 0.655 (95% CI = 0.5416 to 0.7684); (**B**) Salivary miR-29a-3p levels yielded an AUC value of 0.631 (95% CI = 0.5139 to 0.7474); (**C**) Salivary miR-29c-3p levels yielded an AUC value of 0.659 (95% CI = 0.5452 to 0.7733); (**D**) Salivary miR-766-3p levels yielded an AUC value of 0.631 (95% CI = 0.5132 to 0.7484); (**E**) Salivary miR-491-5p levels yielded an AUC value of 0.632 (95% CI = 0.5150 to 0.7497); (**F**) ROC curve analysis for the combination of the five miRNAs yielded an AUC value of 0.754 (95% CI = 0.6524 to 0.8554). Note: ROC, receiver operating characteristic; CRC, colorectal cancer; AUC, area under the ROC curve; CI, confidence interval; miRNAs, microRNAs.

ROC curve analyses were also performed to determine the diagnostic value of the five validated miRNAs (miR-186-5p, miR-29a-3p, miR-29c-3p, miR-766-3p and miR-491-5p) for CRC detection. Importantly, all miRNAs showed good power to discriminate CRC patients from healthy controls with AUC up to 0.659. Importantly, the combined analysis of the five miRNAs showed a higher AUC (0.754), with a sensitivity of 72% and a specificity of 66.67%, differentiating CRC patients from healthy individuals (cut-off value = 0.5132) (Figure 3 and Table S3). Internal validation of the five-miRNA panel showed a bootstrap optimism-corrected AUC value of 0.744 (95% CI = 0.649 to 0.844) for discriminating CRC patients from healthy controls.

### 3.3. Salivary miRNA Levels Were Not Associated with Demographic and Clinicopathological Features 

The relationship between salivary miRNA expression levels and clinicopathological features of CRC patients were also examined. No significant association was found between the five salivary miRNAs and age, lymph node metastasis, distant metastasis, tumor grading, serum CEA levels, TNM staging, tumor location, body mass index, alcohol intake and smoking status, while the levels of miR-186-5p, miR-29a-3p, and miR-29c-3p levels were significantly increased in males (*p* < 0.05). Further, expression levels of salivary miRNAs were slightly increased in non-metastatic patients although no significant differences were observed compared to the late stages (Table S4). Besides, the expression levels of the five validated miRNAs were significantly correlated between them (*p* < 0.0001).

### 3.4. Combination of Salivary miRNA Panel and CEA Improved CRC Detection 

**Figure 4:**
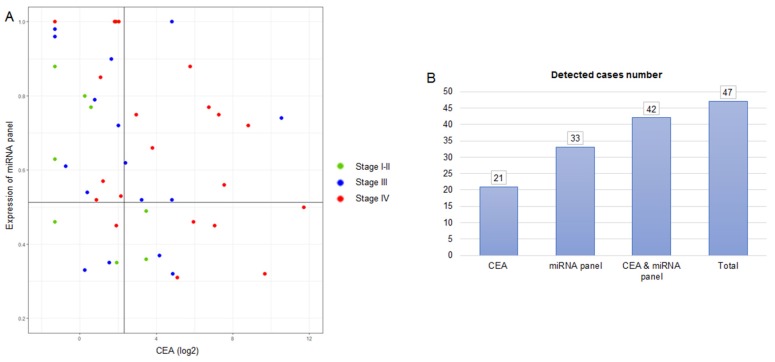
Levels of the five-miRNA panel and serum CEA. (**A**) A combination of the five-miRNA panel and CEA was used to discriminate CRC. The cut-off value for CEA was 5 ng/mL; and the cut-off value for the five-miRNA panel was 0.5132, defined as the model value that provides the highest sensitivity and specificity to discriminate CRC patients from healthy controls. (**B**) Detected cases number using the five-miRNA signature, CEA and their combination. Note: CEA, carcinoembryonic antigen; CRC, colorectal cancer.

We examined the performance of our five-miRNA panel with baseline CEA in 47 CRC patients, a biomarker used for the clinical routine in CRC monitoring. As shown in Figure 4, the five-miRNA panel enabled identifying 33 CRC patients (70.21% sensitivity; cut-off = 0.5132), while CEA levels only enabled identifying 21 CRC patients (44.68% sensitivity; cut-off = 5 ng/mL). Importantly, when we combined the five-miRNA panel and CEA levels, a total of 42 CRC patients were positively detected, showing a diagnostic sensitivity of 89.36%.

### 3.5. Salivary miRNA Panel Showed Value as a Prognosis Marker in Advanced CRC Patients

**Figure 5:**
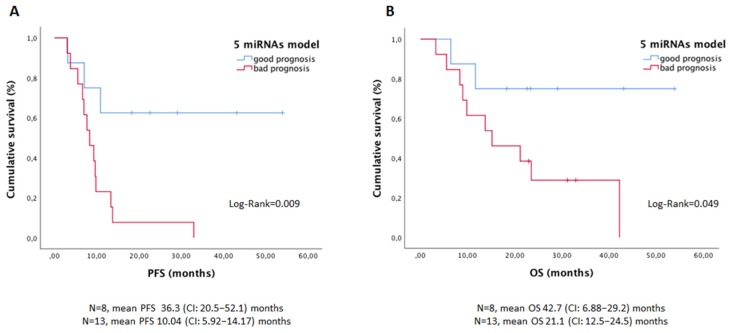
Kaplan–Meier curves to predict PFS (**A**) and OS (**B**) according to the five-miRNA model in stage IV CRC patients. Note: PFS, progression-free survival; OS, overall survival; CI, confidence interval.

In order to determine whether the salivary miRNA signature was associated with patients outcome, survival analyses were performed only in patients with stage IV tumors, because the follow-up period was not enough to determine good/bad prognosis groups in the early stages. A specific risk score was generated with the five miRNAs to discriminate patients with favorable vs. unfavorable evolution, the unfavorable group being those patients progressing before 10 months after sample collection. The univariate Cox regression analysis with the clinicopathological factors and the miRNA model showed a significant higher risk of progression or exitus in patients with higher CEA levels and higher five-miRNA risk score (Figure 5 and Table S5). Importantly, after the multivariate approach, the salivary miRNA model showed independent value in predicting progression-free survival, while serum CEA was a stronger independent predictor for overall survival (Table S6). 

### 3.6. Functional Annotation Analysis of Salivary miRNAs

**Figure 6:**
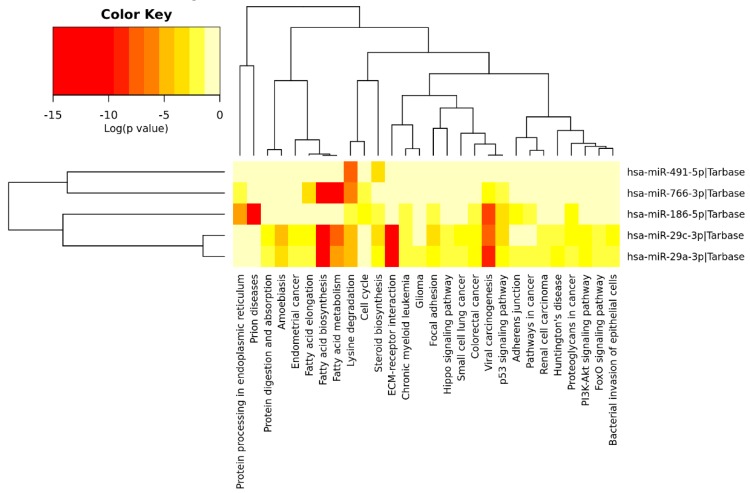
Heatmap of differentially expressed miRNAs vs. significantly enriched functional pathways. In the heatmap, the hottest colors depict higher statistical significance as indicated by the color key at the above. The attached dendrograms on both axes represent hierarchical clustering results for miRNAs and pathways, respectively. Figure was obtained from the output of Diana miRpath V.2.

Significant KEGG pathways in our analysis are presented in Table S7 and Figure 6. This analysis showed three miRNAs (miR-29a-3p, miR-186-5p and miR-29c-3p) directly involved in CRC. Importantly, the five miRNAs (miR-186-5p, miR-29a-3p, miR-29c-3p, miR-766-3p, and miR-491-5p) were linked to the other relevant cell functions and pathways such as p53 or phosphoinositide 3-kinase/protein kinase B (PI3K/AKT), extracellular matrix (ECM) receptor interaction, lipids and amino acids metabolism, cell growth, cell death and cell adhesion. 

## 4. Discussion

Several studies have identified a wide variety of salivary miRNAs as promising non-invasive biomarkers for cancer detection, such as pancreatic, esophageal, prostatic and head and neck cancer [13]. In fact, various saliva-based miRNA panels have been developed for cancer diagnosis. For instance, Xie et al. identified a model composed by miR-3679-5p and miR-940 with 72.5% sensitivity and 70% specificity for differentiating pancreatic cancer patients from controls [19]. Further, in pancreatic cancer, the combination of miR-1246 and miR-4644 yielded 83.3% sensitivity and 92.3% specificity for this tumor diagnosis [20]. These findings demonstrate the potential of saliva as a source of biomarkers for non-oral cancers detection.

Reinforcing the value of saliva characterization to discover new cancer biomarkers, our study described for the first time a saliva-based miRNA signature, composed of miR-186-5p, miR-29a-3p, miR-29c-3p, miR-766-5p and miR-491-5p, that significantly discriminates CRC patients from healthy individuals, reaching 72% sensitivity and 66.67% specificity. Although diagnostic power should be better explored in a future validation study, including a blinded cohort of patients with a larger number of CRC patients at both early and late stages, these results evidenced the interest of the panel. In this sense, bootstrapping analysis highlights the robustness of our five-miRNA panel, showing its potential value for CRC diagnosis. Although few studies have characterized the miRNA expression profile in saliva from different cancer locations, no other study performed wide profiling of salivary miRNAs in CRC patients to date. Actually, the unique study that evaluated saliva as a source of potential biomarkers for diagnostic purposes in CRC was developed by Sazanov et al. [14] These authors found significantly increased salivary expression levels of miR-21 in CRC compared to healthy controls. This miRNA was detected in all cases of CRC included in our discovery phase, showing higher mean expression levels in patients than controls but no significant differences, probably because of the lower number of samples analyzed in our discovery phase. 

Another remarkable finding of our study is that the salivary miRNA panel can be combined with CEA levels, the most routinely used colorectal tumor marker [21], for improving the detection rate of CRC in our study. Of note, CEA levels are mainly used for patients monitoring, but some studies have also reported the value of this marker for CRC cancer detection in combination with other biomarkers [22]. In our cohort of patients, CEA levels were increased only in 44.68%, while our panel was found increased in 70.21%. Importantly, the increment of our salivary miRNA panel or/and CEA levels were found in 89.36% of patients, suggesting that the strategy of combining our salivary-miRNA panel with an established marker for clinical practice could improve CRC detection. In fact, a previous study performed by Vychytilova-Faltejskova et al. described that combining a serum-based four-miRNA panel and CEA levels yielded an increase in CRC detection of 47% compared to using the CEA marker alone [23].

Currently, there are several modalities for CRC screening. However, none has been established as a well-accepted screening tool. Colonoscopy or flexible sigmoidoscopy are invasive and expensive tests, associated with low compliance rates, whereas stool-based tests such as guaiac fecal occult blood and fecal immunochemical tests have low sensitivity in detecting premalignant colorectal adenomas or CRC [24]. Our saliva-based miRNA signature represents a really attractive non-invasive method for CRC diagnosis, with similar rates of detection to other plasma-based tests such as CancerSeek [25]. Importantly, in addition to the value of the salivary miRNA signature identified to detect CRC patients, the signature also showed value in determining those patients with stage IV tumors with poorer prognosis, providing useful clinical information to manage CRC patients. Besides, a potential utility of our salivary miRNA signature, not explored in the present study, could be disease monitoring. For this objective, the panel should be analyzed prospectively in longitudinal saliva samples during disease evolution.

On the other hand, regarding the biological function of these miRNAs, functional analysis revealed that they were implicated in a total of 28 pathways, including 22 cancer-related pathways such as ECM-receptor interaction, p53 and PI3K-Akt signaling pathways, and cell cycle. It is important to highlight that the salivary miRNA panel found in our study is different from all diagnostic models previously reported in blood [26] and tissue [27], although all of them were described as altered miRNAs in CRC. Interestingly, four (miR-186-5p, miR-491-5p, miR-766-3p, and miR-29a-3p) of the five miRNAs from our saliva-based panel have never been described in saliva from patients with cancer and other diseases. Altogether, this suggested that the discovered panel is very specific for detecting CRC. Only miR-29c was found significantly expressed in saliva from patients with unresectable pancreatic cancer, showing 57% sensitivity and 100% specificity for this tumor detection [28]. In fact, miR-29a and miR-29c are members of the miR-29 family, which is known to be associated with tumorigenesis and cancer progression [29]. MiR-29a might act as either an oncogene or tumor suppressor in a wide variety of malignancies [30]. Besides, numerous studies [4,5] have identified the significant upregulation of miR-29a in serum and plasma from CRC patients compared to controls, as we have demonstrated in the current study. On the other hand, miR-29c was described as a tumor suppressor in several human cancers although its role in CRC pathogenesis is unclear. Zhang et al. showed in vitro that miR-29c-3p overexpression decreased significantly the secreted protein acidic and cysteine rich (SPARC) expression, as a potential target in CRC. Further, the silencing of SPARC significantly inhibited proliferation and migration of the CRC cells, while the miR-29c-3p inhibitor promoted it, which indicated that miR-29c-3p acts as a tumor suppressor [31]. Further, miR-29c is thought to be involved in the progression of advanced CRC because its downregulation was observed in primary CRC tissues, while, in metastasis (lung and liver), this miRNA was upregulated [32,33]. Nevertheless, in the present study, no differences were observed for salivary miR-29c-3p between early and late stages. Further, other authors reported circulating miR-29c as a diagnostic and recurrence predictor biomarker for CRC [34,35]. 

The remaining salivary miRNAs from our saliva-based five-miRNA panel (miR-186-5p, miR-491-5p and miR-766-3p) were also previously associated with CRC. Islam et al. found miR-186-5p overexpressed in CRC tissues compared to non-cancer samples and moreover, this expression was correlated with distant metastasis, lymphovascular permeation, and poor prognosis. In addition, the overexpression of miR-186-5p promotes cell proliferation, migration and colony formation by inhibiting the tumor suppressor family with sequence similarity 134, member B (FAM134B) [36]. Recently, Qu et al. found significant overexpression of miR-186-5p in serum samples from CRC patients with lymph node metastasis, which could suggest its involvement in the metastatic process [37]. On the other hand, data regarding miR-491-5p are contradictory. Nakano et al. showed that miR-491 inhibited cell proliferation of CRC cells in vitro and in vivo, inducing apoptosis via the downregulation of B-cell lymphoma extra-large (Bcl-X_L_) [38]. However, recent findings revealed upregulated levels of miR-491 in serum samples from patients with CRC and adenomas compared to control subjects [39]. This result is in line with our results in saliva from CRC patients. Another miRNA involved in the cancer cell biological process is miR-766, which can act as a tumor suppressor or tumor promoter in several cancers [40]. MiR-766 is involved in CRC—its overexpression promotes cell proliferation by repressing SRY-box transcription factor 6 (SOX6), which indicates that miR-766 acts as an oncogene in the development of CRC [41]. Besides, high expression levels of miR-766-3p were found in the plasma of CRC patients compared to healthy individuals [42] but, unlike in our study, no significant differences were observed. 

## 5. Conclusions

Overall, this work represents the first massive characterization of salivary miRNAs in patients with benign and malignant colorectal disease. Our approach identified a set of salivary miRNAs with potential value for discriminating CRC patients from healthy controls and predicting disease outcome in advanced stages non-invasively. These data demonstrated the value of saliva as an important liquid biopsy for CRC detection and prognosis, setting up the basis for a further validation study to really decipher the clinical value of this approach. For this purpose, a large cohort of patients and controls should be evaluated to determine the diagnostic and prognostic accuracy of the panel in a prospective multicenter study including longitudinal samples to also test the panel as a monitoring tool. 

## Supplementary Materials

The following are available online at https://www.mdpi.com/2077-0383/8/12/2029/s1, Figure S1: Study design for CRC salivary miRNAs discovery and validation, Figure S2: Cq values of the selected reference gene, Figure S3: Venn diagram analysis showing overlapping and non-overlapping of differentially expressed miRNAs, Table S1: Clinicopathological characteristics of study participants, Table S2: Candidate reference genes based on expression stability calculated by NormFinder and Coefficient of Variation score, Table S3: Discriminatory power of salivary miRNAs for the detection of CRC, Table S4: Association of salivary miRNA expression levels with clinical variables, Table S5: Univariate Cox regression analysis for clinicopathological parameters and salivary miRNAs in stage IV CRC patients included in the study, Table S6: Multivariate Cox regression analysis for clinicopathological parameters and salivary miRNAs in stage IV CRC patients included in the study, and Table S7: Significantly enriched signaling pathways in our five-miRNA salivary panel (*p* < 0.05).
