# Supplementary material for: A Novel Saliva-Based miRNA Signature for Colorectal Cancer Diagnosis
Source: J Clin Med. 2019 Nov 20;8(12):2029. doi: 10.3390/jcm8122029 (PMC6947363; doi:10.3390/jcm8122029)
Supplement: Supplementary file 1 [file jcm-08-02029-s001.pdf]

A novel Saliva-based miRNA Signature for Colorectal Cancer Diagnosis

Óscar Rapado-González, Blanca Majem, Ana Álvarez-Castro, Roberto Díaz-Peña, Alicia Abalo, Leticia Suárez-Cabrera, Antonio Gil-Moreno, Anna Santamaría, Rafael López-López, Laura Muinelo-Romay, María Mercedes Suarez-Cunqueiro

Supplementary Methods

Saliva collection and processing

Saliva samples were collected and processed as described previously Majem et al. (Majem et al. 2017). Unstimulated whole saliva samples were collected from the participants between 9 and 10 am, before any therapeutic procedures. Subjects refrained from eating, drinking and oral hygiene procedures for at least 1 hour before the collection. Subjects rinsed their mouth with distilled water to minimize contamination of the salivary samples. Five min after the oral rinsing, the participant start to spit into a 50-mL Falcon tube kept on ice. As a minimum, five milliliters of saliva was collected from each participant. Immediately after collection, salivary samples were centrifuged at 2,600 x *g* for 15 min at 4°C to remove cellular components. Saliva supernatant was then separated from the pellet and 1 µL per milliliter of supernatant saliva of RNase inhibitor (SUPERase-In, AM2694, Ambion, Life Technologies) was added. All samples were aliquoted in 1,200 µL and stored at –80°C prior to assay. Salivary samples were randomized for RNA extractions, RT reactions and RTqPCR analyses in all cases.

Saliva RNA extraction

At first, frozen saliva samples were thawed thoroughly on ice and centrifuged 10,000 x *g* at 4°C for 5 min to eliminate cell debris saliva. Then, cell-free saliva was separated into 2 eppendorf of 250 µL-saliva which were lysed in 750 µL of Trizol LS Reagent (10296-028, Ambion, Life Technologies). Thereafter, 200 µL chloroform was added to the denatured saliva and mixed by vortex for 30 s, followed by incubation for 5 min at room temperature. The addition of chloroform causes phase separation where protein is extracted to the organic phase, DNA resolves at the interface, and RNA remains in the aqueous phase. Total RNA was eluted from the spin column membrane in 60 µL pre-heated RNA-free water (50ºC) and DNase treatment (DNase, Rocher) was used to remove contaminating DNA during RNA extraction. After RNA precipitation, the final RNA was suspended in 10 µL pre-heated RNA-free water (50ºC), then incubated for 5 min at 55ºC and RNA samples were stored at -80ºC for further analyses.

Salivary miRNA discovery profiling with TaqMan Low Density Arrays

This miRNA microarray assay was performed using 3 μL (1-350 ng) of total RNA from each sample, according to the manufacture's protocol. From each sample, 3 μL (1-350 ng) of total RNA were reverse-transcribed using Megaplex™ RT primer pool (pool A v2.1 and pool B v3.0, Applied Biosystems) and TaqMan® MicroRNA reverse transcription kit (Applied Biosystems). In order to increase the sensitivity, cDNA was preamplified using Megaplex™ PreAmp primer pool (pool A v2.1 and pool B v3.0, Applied Biosystems) and TaqMan® Preamp Master Mix (Applied Biosystems). Then, samples were mixed with TaqMan Universal PCR Master Mix, No AmpErase® UNG, 2X (Applied Biosystems), and applied to the TLDA cards. The 8 ports on each array card were loaded with 100 μL reaction followed by brief centrifugation and sealing. RT-qPCR was performed at 7900HT Fast Thermocycler System. After real-time quantitative reverse-transcriptase polymerase chain reaction (RT-qPCR) raw data were exported to the ExpressionSuite software (v.1.1, Applied Biosystem) for performing global normalization and quality control. Threshold and baseline were automatically

calculated for each assay, and a global normalization was performed as recommended for large scale miRNA expression profiling (Mestdagh et al. 2009). MiRNAs with significantly different levels ($P < 0.05$) between CRC and healthy controls samples, a 1.5-fold change (up or down) and positive expression in at least 70% of tumor samples was considered as candidates to be confirmed in a larger cohort of samples.

MiRNA expression analysis using RT-qPCR

A total volume of 3 µL of each sample was reverse transcribed using TaqMan MicroRNA Reverse Transcription Kit (Applied Biosystems, Thermo Fischer, Baltics, UAB) in a total volume of 15 µL with the following thermal-cycling conditions: 16 ºC for 30 min, 42ºC for 30 min, 85 ºC for 5 min and then storing at 4 ºC. Then, 2.5 µL of cDNA was pre-amplified using TaqMan® PreAmp Master Mix (Applied Biosystems, Thermo Fischer, Foster City, CA, USA) with the following thermal-cycling conditions: 95ºC for 10 min, 55ºC for 2 min, 72ºC for 2 min, 12 cycles at 95 ºC for 15 s and 60 ºC for 4 min, then 99.9ºC for 10 min. Then samples were stored at -20ºC. RT-qPCR analyses were conducted in 96-well plates (Applied Biosystems) in a reaction volume of 15 µL using TaqMan® Gene Expression Master Mix (Applied Biosystems, Thermo Fischer). All RT-qPCR reactions were run in the StepOne Plus system (Applied Biosystems) with the following cycling conditions: 50ºC for 2 min, 95ºC for 10 min, followed by 40 cycles of 95ºC for 15 s and 60ºC for 1 min. All reactions were performed in duplicates for each sample, and a no-template control was used as a negative control. Cycle quantification (Cq) values were calculated using StepOne Software v2.3 (Applied Biosystems) for target miRNAs and endogenous normalizers.

Selection of internal controls for quantification of salivary miRNAs

For validating the selected miRNAs, miRNA profiling data of the discovery cohort was used to choose an appropriate endogenous control based on the following criteria: (a) Relative quantification close to 1:1 (where the healthy group is the reference biological group); (b) miRNA detected in more than 90% of all replicates of all samples; (c) amplification score above 1.24 and (d) no differential expression between cancer and control samples ($P > 0.05$). As U6 small nuclear RNA, which is normally used as a reference gene for miRNAs expression analyses, was not uniformly expressed in samples after microarray analysis, 2 miRNAs (miR-1274B and miR-193b-3p) were selected as candidate reference genes because there were homogeneously expressed between groups in terms of Cq values between groups (miR-1274B ($P = 0.279$) and miR-193b-3p ($P = 0.584$) and their levels appear to be stable across the whole cohort. The stability of selected reference genes was calculated using two different algorithms, NormFinder for R (version 5, 2015-01-05; https://moma.dk/normfinder-software) and Coefficient of Variation score. MiR-193b-3p expression levels showed the lowest intragroup and intergroup variability (Supplementary Table S2). In order to further validate the stability of miR-193b-3p and miR-1274B, we analyzed the expression patterns in 45 saliva samples (30 CRC and 15 HC). Using the Cq values of each validated reference gene, there was no evidence for differential expression of miR-193b-3p whereas salivary expression levels of miR-1274B were significantly different between CRC patients and healthy individuals ($P = 0.0276$). Therefore, miR-193b-3p was chosen as an internal control to perform all the RT-qPCR analyses due to its high stability among samples (Figure S2).

References

Majem B, Li F, Sun J, Wong DT. RNA Sequencing analysis of salivary extracellular RNA. Methods Mol Biol 2017;1537:17–36.

Mestdagh P, Van Vlierberghe P, De Weer A, Muth D, Westermann F, Speleman F, et al. A novel and universal method for microRNA RT-qPCR data normalization. Genome Biol 2009;10:R64.

Table S1. Clinicopathological characteristics of study participants.

	Discovery Phase		Validation Phase		
	HC (*n* = 10)	**CRC (*n* = 14)**	**HC (*n* = 37)**	**CRC (*n* = 51)**	**A (*n* =19)**
Age (years)					
Mean ± SD	71.90 ± 12.39	67.21 ± 11.27	62.81 ± 12.19	66.27 ± 11.16	63.95 ± 8.13
Median	76.50	65.50	62	66	60
Range	53-87	50-85	38-86	46-85	52-80
Gender					
Male	3 (30%)	10 (71.4%)	19 (51.4%)	33 (64.7%)	14 (73.7%)
Female	7 (70%)	4 (28.6%)	18 (48.6%)	18 (35.3%)	5 (26.3%)
TNM status					
I	–	2 (14.3%)	–	6 (11.8%)	–
II	–	2 (14.3%)	–	3 (5.9%)	–
III	–	3 (21.4%)	–	21 (41.2%)	–
IV	–	7 (50%)	–	21 (41.2%)	–
Tumor (T)					
Tx	–	3 (21.4%)	–	5 (9.8%)	–
T1	–	–	–	–	–
T2	–	2 (14.3%)	–	6 (11.8%)	–
T3	–	5 (35.7%)	–	25 (49.0%)	–
T4	–	4 (28.6%)	–	15 (29.4%)	–
Regional lymph node status (N)					
N0	–	4 (28.6%)	–	11 (21.6%)	–
N1	–	5 (35.7%)	–	23 (45.1%)	–
N2	–	2 (14.3%)	–	12 (23.5%)	–
Nx	–	3 (21.4%)	–	5 (9.8%)	–
Distant metastasis status (M)					
M0	–	7 (50%)	–	30 (58.8%)	–
M1	–	7 (50%)	–	21 (41.2%)	–
Tumor location					
Cecum	–		–	2 (3.9%)	–
Colon	–	2 (14.3%)	–	11 (21.6%)	14 (73.7%)
Sigma	–	6 (42.9%)	–	15 (29.4%)	3 (15.8%)
Rectum-sigma	–	–	–	3 (5.9%)	-
Rectum	–	6 (42.9%)	–	20 (39.2%)	2 (10.5%)
Histological types					
Adenocarcinoma	–	14 (100%)	–	48 (94.1%)	-
Mucinous adenocarcinoma	–	–	–	1 (2%)	-
Signet ring cell	–	–	–	2 (3.9%)	-
Tubular adenoma	–	–	–	–	12 (63.2%)
Tubulovillous adenoma	–	–	–	–	7 (36.8%)
Unknown	–	–	–	–	–
Grading					
Low grade	–	13 (92.9%)	–	39 (76.5%)	15 (78.9%)
High grade	–	1 (7.1%)	–	4 (7.8%)	4 (12.5%)
Unknown	–	–	–	8 (15.7%)	–
Smoking status					
Non-smoker	7 (70%)	5 (35.7%)	20 (54.1%)	22 (43.1%)	6 (31.6%)
Ex-smoker	2 (20%)	5 (35.7%)	13 (35.1%)	21 (41.2%)	7 (36.8%)
Smoker	1 (10%)	4 (28.6%)	4 (10.8%)	8 (15.7%)	6 (31.6%)
Alcohol intake					
Never	7 (70%)	11 (78.6%)	22 (59.5%)	31 (60.8%)	10 (52.6%)

Current	3 (30%)	3 (21.4%)	15 (40.5%)	20 (39.2%)	9 (47.4%)
BMI					
Normal	–	–	–	4 (7.8%)	–
Pre-obesity	–	9 (64.3%)	–	27 (52.9%)	–
Obesity	–	3 (21.4%)	–	14 (27.5%)	–
Unknown	–	2 (14.3%)	–	6 (11.8%)	–
CEA (ng/ml)					
Mean ± SD	–	24.59 ± 36.65	–	146.37 ± 530.70	–
Median	–	6.05	–	4.00	–
Range	–	1.20-131.90	–	0.40-3330.90	–

HC, healthy controls; A, adenomas; CRC, colorectal cancer; BMI, body mass index; CEA, carcinoembryonic antigen; SD, standard deviation.

Table S2. Candidate reference genes based on expression stability calculated by NormFinder and Coefficient of Variation score.

	RQ	Detection*	Cq mean (tumor/control)	Cq median (tumor/control)	*P* Value	NormFinder stability value	CV	SD
miR-193b-3p	1:1.017	100%	27/27.71	26.71/27.49	0.5387	0.05	0.11	3.20
miR-1274B	1:1.046	95.83%	24.01/25.23	23.86/25.65	0.1450	0.06	0.14	3.54

RQ, relative quantification; CV, coefficient of variation; SD, standard deviation. *Percentage of samples where the miRNA was detected.

Table S3. Discriminatory power of salivary miRNAs for the detection of CRC.

CRC vs. HC	AUC	95% CI	S (%)	95% CI	Sp (%)	95% CI	cut-off point	*P* Value
miR-186-5p	0.6550	0.5416 to 0.7684	64.71	50.07 to 77.57	54.05	36.92 to 70.51	1.096	0.013
miR-29a-3p	0.6306	0.5139 to 0.7474	64.71	50.07 to 77.57	56.76	39.49 to 72.90	0.9864	0.037
miR-29c-3p	0.6592	0.5452 to 0.7733	64.71	50.07 to 77.57	62.16	44.76 to 77.54	1.184	0.011
miR-766-3p	0.6308	0.5132 to 0.7484	64	49.19 to 77.08	62.16	44.76 to 77.54	1.477	0.037
miR-491-5p	0.6324	0.5150 to 0.7497	70.59	56.17 to 82.51	50	32.92 to 67.08	1.126	0.036
5-miRNA panel	0.7539	0.6524 to 0.8554	72	57.51 to 83.77	66.67	49.03 to 81.44	0.5132	<0.001

CRC, colorectal cancer; HC, healthy controls; AUC, area under the ROC curve; Se, sensitivity; Sp, specificity; CI, confidence interval.

Table S4. Association of salivary miRNAs expression levels with clinical variables.

Characteristics		miR-186-5p		miR-29a-3p		miR-29c-3p		miR-491-5p		miR-766-3p	
	Total (%)	Mean ± SD	*P* Value	Mean ± SD	*P* Value	Mean ± SD	*P* Value	Mean ± SD	*P* Value	Mean ± SD	*P* Value
Gender											
Male	33 (64.7)	3.81 ± 3.04	**0.016**	3.36 ± 3.05	**0.030**	3.12 ± 2.62	**0.026**	2.94 ± 2.23	0.056	3.19 ± 2.58	0.055
Female	18 (35.3)	1.98 ± 2.55		2.14 ± 3.07		2.24 ± 3.46		2.48 ± 4.16		2.12 ± 2.52	
Age (years)											
≥ 65	28 (54.9)	2.75 ± 2.88	0.167	2.29 ± 2.38	0.140	2.20 ± 2.06	0.191	2.03 ± 1.81	0.072	2.39 ± 2.59	0.085
< 65	23 (45)	3.66 ± 3.09		3.71 ± 3.67		3.55 ± 3.66		3.69 ± 3.88		3.29 ± 2.56	
Metastasis											
Negative	30 (58.8)	3.24 ± 3.01	0.688	3.02 ± 3.09	0.688	2.90 ± 3.05	0.730	3.05 ± 3.54	0.646	3.05 ± 2.94	0.716
Positive	21 (41.2)	3.05 ± 3.02		2.80 ± 3.14		2.68 ± 2.84		2.40 ± 2.09		2.46 ± 2.02	
Nodes											
Negative	11 (21.6)	2.99 ± 2.91	0.652	3.10 ± 3.51	0.652	3.23 ± 4.10	0.690	2.71 ± 2.72	0.728	2.71 ± 2.37	0.990
Positive	35 (68.6)	3.37 ± 3.17		3.06 ± 3.13		2.87 ± 2.70		2.99 ± 3.30		2.93 ± 2.83	
Grading											
Low grade	39 (76.5)	3.46 ± 3.04	0.702	3.19 ± 3.10	0.531	3.06 ± 2.99	0.531	3.00 ± 3.20	0.856	2.96 ± 2.69	0.605
High grade	4 (7.8)	2.48 ± 1.92		1.77 ± 1.60		1.76 ± 1.35		2.52 ± 2.18		2.86 ± 0.98	
TNM stage											
I	6 (11.8)	2.38 ± 2.06	0.839	2.82 ± 3.76	0.889	3.48 ± 5.21	0.939	2.25 ± 2.26	0.739	2.35 ± 1.98	0.839
II	3 (5.9)	3.88 ± 4.33		3.60 ± 4.14		2.65 ± 2.78		4.09 ± 4.04		4.05 ± 3.36	
III	21 (41.2)	3.40 ± 3.15		3.00 ± 2.92		2.77 ± 2.41		3.13 ± 3.86		3.12 ± 3.20	
IV	21 (41.2)	3.05 ± 3.02		2.80 ± 3.14		2.68 ± 2.84		2.40 ± 2.09		2.46 ± 2.02	
Early (I-II)	9 (17.6)	2.88 ± 2.86	0.780	3.08 ± 3.64	0.799	3.20 ± 4.37	0.743	2.86 ± 2.85	0.971	2.92 ± 2.45	0.728
Late (III-IV)	42 (82.4)	3.22 ± 3.05		2.90 ± 3.00		2.73 ± 2.60		2.76 ± 3.09		2.78 ± 2.65	
Tumor location											
Left	40 (78.4)	3.16 ± 2.90	0.732	3.01 ± 3.08	0.602	2.87 ± 2.96	0.585	2.86 ± 3.18	0.713	2.86 ± 2.63	0.979
Right	9 (17.6)	3.40 ± 3.66		2.79 ± 3.47		2.77 ± 3.23		2.43 ± 2.41		2.93 ± 2.71	
CEA (ng/mL)											
≥5	21 (44.7)	2.53 ± 2.84	0.441	2.38 ± 2.37	0.983	2.13 ± 1.98	0.748	2.83 ± 3.85	0.847	2.45 ± 2.54	0.559
<5	26 (55.3)	3.39 ± 3.05		3.16 ± 3.56		3.20 ± 3.57		2.78 ± 2.48		2.75 ± 2.32	
BMI											
Normal	4 (7.8)	5.39 ± 4.38	0.447	5.09 ± 4.21	0.464	4.50 ± 3.48	0.496	6.80 ± 7.66	0.346	4.21 ± 4.66	0.756

Pre-obesity	27 (52.9)	3.27 ± 3.05		2.98 ± 3.07		2.95 ± 3.20		2.66 ± 2.26		2.91 ± 2.64	
Obesity	14 (27.5)	2.36 ± 2.38		2.10 ± 2.73		2.12 ± 2.47		2.10 ± 2.02		2.37 ± 2.17	
Alcohol intake											
Never	31 (60.8)	2.70 ± 2.93	0.086	2.70 ± 3.42	0.067	2.64 ± 3.36	0.086	2.82 ± 3.61	0.263	2.69 ± 2.96	0.153
Current	20 (39.2)	3.87 ± 3.00		3.28 ± 2.51		3.07 ± 2.20		2.72 ± 1.83		3.00 ± 1.888	
Smoking status											
Non-smoker	22 (43.1)	2.24 ± 2.39	0.052	1.80 ± 1.88	0.078	1.78 ± 1.73	0.082	1.69 ± 1.43	0.058	2.08 ± 1.70	0.116
Ex-smoker	21 (41.2)	4.54 ± 3.45		4.18 ± 3.66		3.73 ± 3.08		4.13 ± 4.07		3.91 ± 3.30	
Smoker	8 (15.7)	2.08 ± 1.71		2.75 ± 3.21		3.23 ± 4.43		2.23 ± 1.40		1.80 ± 1.05	

BMI, body mass index; SD, standard deviation; CEA, carcinoembryonic antigen.

Table S5. Univariate Cox regression analysis for clinicopathological parameters and salivary miRNAs in stage IV CRC patients included in the study.

	PFS		OS	
	HR (95% CI)	*P* Value	HR (95% CI)	*P* Value
Gender (male vs. female)	0.65 (0.20–2.07)	0.46	0.79 (0.20–3.05)	0.73
Age (<65 vs. ≥65 years)	1.45 (0.52–4.05)	0.47	1.12 (0.35–3.60)	0.84
Lymph node metastasis (no vs. yes)	0.48 (0.06–3.77)	0.47	0.03 (0.01–52.64)	0.14
Liver metastasis (no vs. yes)	0.74 (0.16–3.37)	0.72	1.19 (0.15–9.49)	0.86
CEA serum levels (<5 vs. ≥5 ng/mL)	4.44 (1.3–14.54)	**0.008**	5.22 (1.13–24.1)	**0.018**
5miRNAs-panel model (good vs. bad prognosis)	4.73 (1.31–17.01)	**0.009**	4.13 (0.89–19.15)	**0.049**

HR represent the risk of the bad prognosis group vs. the good prognosis group. PFS, progression-free survival; OS, overall survival; HR, hazard ratio; CI, confidence interval.

Table S6. Multivariate Cox regression analysis for clinicopathological parameters and salivary miRNAs in stage IV CRC patients included in the study.

	PFS	
	HR (95% CI)	*P* Value
CEA serum levels (<5 vs. ≥5 ng/mL)	2.61 (0.39–17.2)	0.31
5miRNAs-panel model (good vs. bad prognosis)	4.73 (1.3–17.01)	**0.017**
	OS	
	HR (95% CI)	*P* Value
CEA serum levels (<5 vs. ≥5 ng/mL)	5.22 (1.13–24.09)	**0.034**
5miRNAs-panel model (good vs. bad prognosis)	1.41 (0.15–12.77)	0.75

HR represent the risk of the bad prognosis group vs. the good prognosis group. PFS, progression-free survival; OS, overall survival; HR, hazard ratio; CI, confidence interval.

Table S7. Significantly enriched signaling pathways in our five miRNAs salivary panel (*P* < 0.05).

KEGG pathway maps	Enriched pathways (nº of miRNAs implicated)	Adjusted *P* Values
Environmental Information Processing		
Signaling molecules and interaction	ECM-receptor interaction (2)	<1.00E-325
Signal transduction	PI3K-Akt signaling pathway (2)	0.00175845
	Hippo signaling pathway (2)	0.01932169
	FoxO signaling pathway (2)	0.03226584
Metabolism		
Lipid metabolism	Fatty acid biosynthesis (3)	<1.00E-325
	Fatty acid metabolism (3)	<1.00E-325
	Steroid biosynthesis (4)	1.92E-08
	Fatty acid elongation (3)	0.00012716

Amino acid metabolism	Lysine degradation (5)	<1.00E-325
Cellular Processes		
Cell growth and death	p53 signaling pathway (4)	5.17E-09
	Cell cycle (2)	0.00013274
Cellular community - eukaryotes	Focal adhesion (3)	3.02E-05
	Adherens junction (2)	0.00497083
Human Diseases		
Cancer: overview	Viral carcinogenesis (4)	<1.00E-325
	Proteoglycans in cancer (3)	0.00227843
	Pathways in cancer (2)	0.04253708
Neurodegenerative disease	Prion diseases (1)	2.22E-16
	Huntington disease (2)	0.00876501
Infectious disease: parasitic	Amoebiasis (2)	2.23E-05
Cancer: specific types	Colorectal cancer (3)	0.0001118
	Chronic myeloid leukemia (3)	0.00426377
	Small cell lung cancer (2)	0.00475059
	Endometrial cancer (2)	0.02146469
	Glioma (1)	0.03492608
	Renal cell carcinoma (2)	0.04238292
Infectious disease: bacterial	Bacterial invasion of epithelial cells (2)	0.00686747
Genetic Information Processing		
Folding, sorting and degradation	Protein processing in endoplasmic reticulum (2)	6.77E-05
Organismal Systems		
Digestive system	Protein digestion and absorption (2)	0.04884862

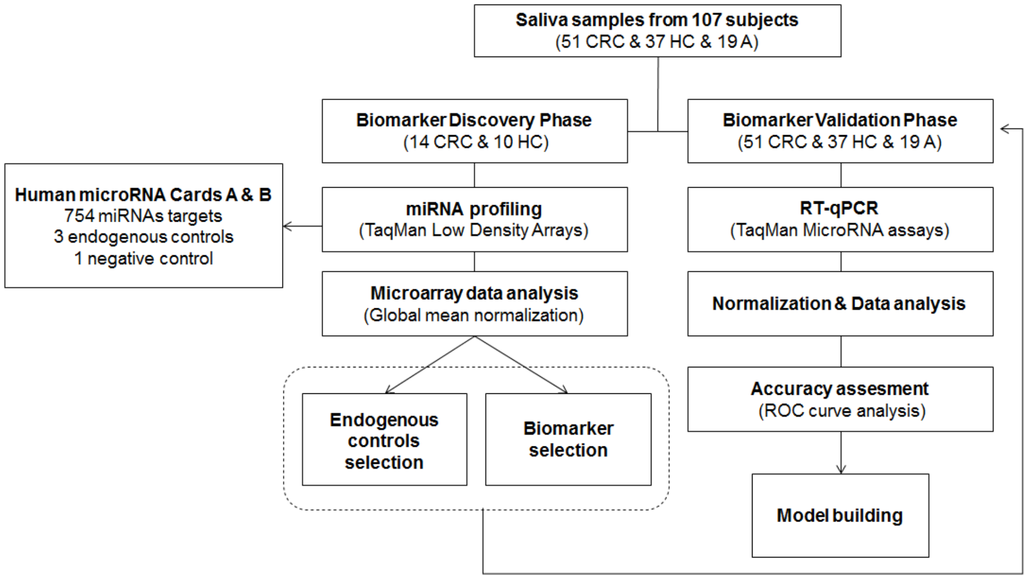

Figure S1. Study design for CRC salivary miRNAs discovery and validation. HC, healthy controls; A, adenomas; CRC, colorectal cancer.

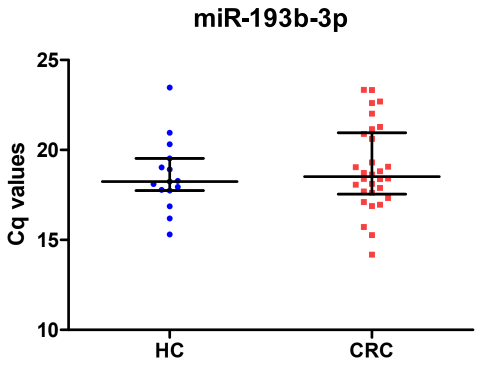

Figure S2. Cq values of the selected reference gene. No significant difference was found between the groups ($P > 0.05$, Mann Whitney *U* test). HC, healthy controls; CRC, colorectal cancer.

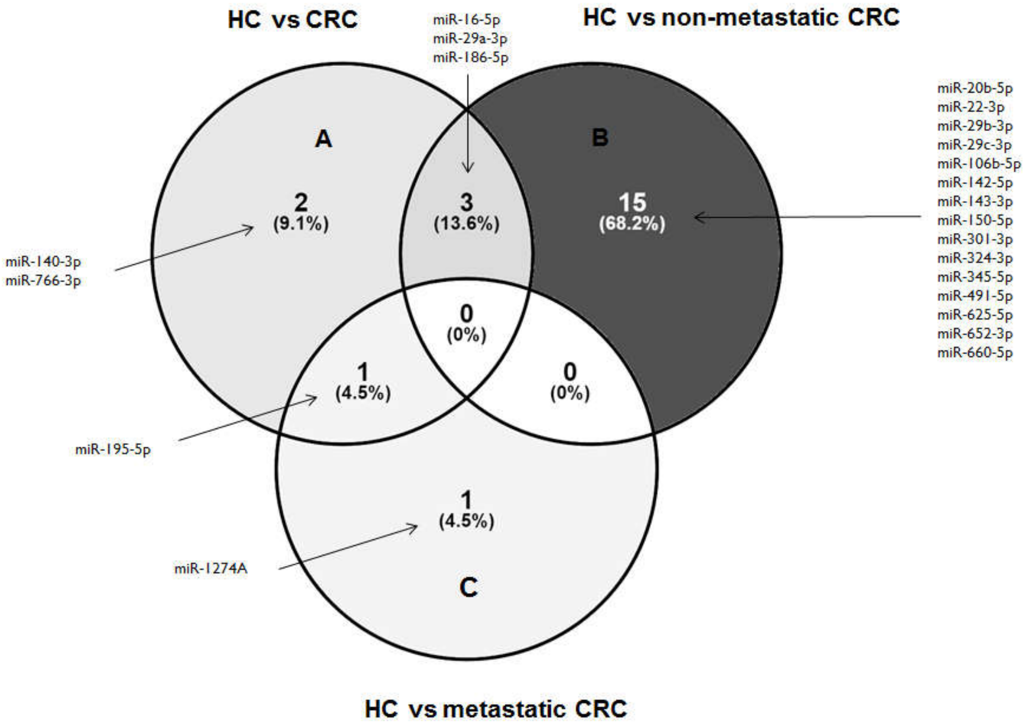

Figure S3. Venn diagram analysis showing overlapping and non-overlapping of differentially expressed miRNAs. (A) Healthy controls and CRC patients. (B) Healthy controls and non-metastatic CRC patients. (C) Healthy controls and metastatic CRC patients.
